# Tracking Improvement in Simulated Marine Biogeochemistry Between CMIP5 and CMIP6

**DOI:** 10.1007/s40641-020-00160-0

**Published:** 2020-08-18

**Authors:** Roland Séférian, Sarah Berthet, Andrew Yool, Julien Palmiéri, Laurent Bopp, Alessandro Tagliabue, Lester Kwiatkowski, Olivier Aumont, James Christian, John Dunne, Marion Gehlen, Tatiana Ilyina, Jasmin G. John, Hongmei Li, Matthew C. Long, Jessica Y. Luo, Hideyuki Nakano, Anastasia Romanou, Jörg Schwinger, Charles Stock, Yeray Santana-Falcón, Yohei Takano, Jerry Tjiputra, Hiroyuki Tsujino, Michio Watanabe, Tongwen Wu, Fanghua Wu, Akitomo Yamamoto

**Affiliations:** 1 CNRM, Université de Toulouse, Météo-France, CNRS, Toulouse, France; 2 National Oceanography Centre, European Way, Southampton, SO14 3ZH UK; 3 LMD-IPSL, Ecole Normale Supérieure / Université PSL, CNRS, Ecole Polytechnique, Sorbonne Université, Paris, PSL University, Paris, France; 4 School of Environmental Sciences, University of Liverpool, Liverpool, UK; 5 LOCEAN Laboratory, Sorbonne Université-CNRS-IRD-MNHN, Paris, France; 6 Canadian Centre for Climate Modelling and Analysis, Victoria, BC Canada; 7 NOAA/Geophysical Fluid Dynamics Laboratory, Princeton, NJ USA; 8 LSCE-IPSL, Université Paris Saclay, Gif-sur-Yvette, France; 9 Max Planck Institute for Meteorology, Hamburg, Germany; 10 National Center for Atmospheric Research, Boulder, CO USA; 11 JMA Meteorological Research Institute, Tsukuba, Japan; 12 NASA Goddard Institute for Space Studies, New York, USA; 13 NORCE Climate, Bjerknes Centre for Climate Research, Bergen, Norway; 14 Present Address: Los Alamos National Laboratory, Los Alamos, NM USA; 15 Research Center for Environmental Modeling and Application, Japan Agency for Marine-Earth Science and Technology (JAMSTEC), Yokohama, Japan; 16 Beijing Climate Center, China Meteorological Administration, Beijing, China

**Keywords:** Marine Biogeochemistry, CMIP5, CMIP6, Biogeochemistry-Climate Feedbacks, Model Performance

## Abstract

**Purpose of Review:**

The changes or updates in ocean biogeochemistry component have been mapped between CMIP5 and CMIP6 model versions, and an assessment made of how far these have led to improvements in the simulated mean state of marine biogeochemical models within the current generation of Earth system models (ESMs).

**Recent Findings:**

The representation of marine biogeochemistry has progressed within the current generation of Earth system models. However, it remains difficult to identify which model updates are responsible for a given improvement. In addition, the full potential of marine biogeochemistry in terms of Earth system interactions and climate feedback remains poorly examined in the current generation of Earth system models.

**Summary:**

Increasing availability of ocean biogeochemical data, as well as an improved understanding of the underlying processes, allows advances in the marine biogeochemical components of the current generation of ESMs. The present study scrutinizes the extent to which marine biogeochemistry components of ESMs have progressed between the 5th and the 6th phases of the Coupled Model Intercomparison Project (CMIP).

**Electronic supplementary material:**

The online version of this article (10.1007/s40641-020-00160-0) contains supplementary material, which is available to authorized users.

## Introduction

Marine biogeochemistry plays a key role in the Earth system. By regulating the exchange of CO2 and other climatically active gases with the atmosphere [1], it is involved in a large range of climate feedbacks [2]. As a result, changes in ocean biogeochemistry can have important consequences for climate [3–5]. Marine biogeochemistry is also deeply interwoven with the functioning of marine ecosystems and ultimately food webs [6–8]. Marine ecosystems are affected by anthropogenic environmental change [9–11], particularly through climate-induced changes in physical properties and CO_2_-induced ocean acidification [12–16]. Understanding and quantifying the response of ocean biogeochemistry to global changes, as well as its role in Earth system feedbacks [12, 17], are essential to improve our capacity to project ecosystem services and climate change in this century and beyond.

In this context, ocean biogeochemical models are acknowledged as powerful tools to study the ocean carbon cycle and its response to past and future climate and chemical changes [2]. Since the pioneering assessment of anthropogenic carbon uptake by the ocean by Maier-Reimer and Hasselmann [18] and Sarmiento et al. [19], and the Ocean Carbon Model Intercomparison Project (OCMIP) of Orr et al. [20], ocean biogeochemical models have been successfully integrated in many Earth system models (e.g. [21–31]).

Over the last few decades, the results from ocean biogeochemical models running within ESMs have increasingly been used to drive research on the carbon cycle. Their results have supported the assessment of carbon cycle feedbacks [32–35] and have improved the understanding of mechanisms behind the near-linear transient climate response to cumulative CO_2_ emissions [36]. Consequently, they have helped determine the change in carbon budgets that is compatible with a given level of warming since pre-industrial times. Ocean biogeochemical models have also been used to investigate potential geoengineering solutions to climate change such as solar radiation management [37–39], ocean fertilization [40–47], alkalinity addition [48–52] and reversibility experiments (e.g. [53, 54]).

Recent advances in marine ecosystem modelling have also led to diversification in the use of ocean biogeochemistry models within ESMs to study a wide range of potential impacts [55–58]. These research activities are now grouped under the umbrella of the Inter-Sectoral Impact Model Intercomparison Project (ISIMIP), with the FishMIP initiative being a specific example for fisheries impacts [59, 60].

Over recent years, models are increasingly being used in a semi-operational mode to aid with investigations of the predictability of key policy-relevant ocean biogeochemistry fields (e.g. net primary productivity, ocean acidity, ocean carbon uptake) [61–67]. Because of their close relationship with important living marine resources, skillful predictions of these properties have led to ocean biogeochemistry models being recognized as valuable tools when developing environmental policies (e.g. [68]) or designing fisheries management [64, 65, 69].

Because this large array of applications goes well beyond the conventional scientific investigation of the ocean carbon cycle, marine biogeochemical models have been developed in a number of directions over recent years. These developments are generally supported by progress in process understanding, which in turn is driven by an increasing number of observational databases [70–72]. However, from one generation to another, the development of marine biogeochemical models is driven not only by common scientific considerations but also by the internal priorities of individual modelling groups. As a consequence, it is difficult to anticipate how far the representation of marine biogeochemistry within the current generation of Earth system models differs from—and has improved upon—the previous one.

The present study maps the changes or updates in ocean biogeochemistry components that have arisen between CMIP5 and CMIP6 and assesses how far these have led to actual improvements in model skill against present-day observations. Overall, our assessment demonstrates that the simulated mean state of ocean biogeochemistry models in CMIP6 is more realistic than that produced by their CMIP5 analogues in many aspects, but that it remains difficult to clearly identify which changes in a given ocean biogeochemistry model are responsible for these improvements.

## Mapping Changes or Updates in Ocean Biogeochemistry

In this section, we review the changes or updates implemented by participating modelling groups. The following method was employed to collect relevant model details as shown in Table 1. First, all of the modelling groups contributing both to CMIP5 and CMIP6 were approached. Next, a questionnaire in the form of a spreadsheet was proposed and developed. This sought details around (1) model resolution, (2) complexity in marine biology, (3) the representation of bacteria, (4) internal physiology, (5) organic matter cycling, (6) sediments, (7) nutrients and elemental cycling, (8) the level of interactions with the other components of the Earth system and (9) modelling approaches including spin-up protocols and tuning/calibration. The latter includes external inputs/outputs and biophysical interactions. The resulting master table of model properties is provided in Supplementary materials (Table S1).

**Table 1:**
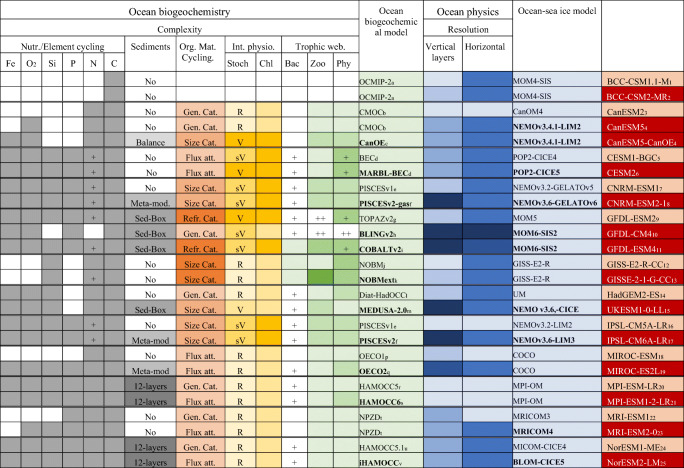
Overview of the ocean and marine biogeochemical components of Earth system models as used in CMIP5 and CMIP6. The names of the ESM are given in the first line of the table where the CMIP6 ESMs are given in red cells and the CMIP5 predecessors are given in pink cells. The complexity of the marine biogeochemical models is described using (i) the trophic web, the number of living species or phytoplankton functional types; (ii) the internal physiology, the stoichiometry and the representation of internal photosynthetic pigment; (iii) the organic matter cycling, the number of organic carbon pools and their representation; (iv) the representation of marine sediments and (v) the nutrient cycling: the number of nutrients and the representation of oxygen and iron cycling

The reference paper of the reviewed ESMs is ^1^Wu et al. [31], ^2^Wu et al. [73], ^3^Arora et al. [22], ^4^Swart et al. [74], ^5^Lindsay et al. [27], ^6^Danabasoglu et al. [75], ^7^Séférian et al. [29, 76], ^8^Séférian et al. [77], ^9^Dunne et al. [25], ^10^Held et al. [78], ^11^Krasting et al. [79], ^12^Romanou et al. [28], ^13^Ito et al. [80], ^14^Jones et al. [81], ^15^Sellar et al. [82], ^16^Dufresne et al. [24], ^17^Boucher et al. [83], ^18^Watanabe et al. [30], ^19^Hajima et al. [84], ^20^Giorgetta et al. [26], ^21^Mauritsen et al. [85], ^22^Adachi et al. [21], ^23^Yukimoto et al. [86], ^24^Bentsen et al. [23], ^25^Seland et al. [87]. The reference paper of marine biogeochemical model is ^a^http://www.ipsl.jussieu.fr/OCMIP/phase2, ^b^Zahariev et al. [88], ^c^Hayashida et al. [89], ^d^Moore et al. [90], ^e^Aumont and Bopp [40, 41], ^f^Aumont et al. [91], ^g^Dunne et al. [25], ^h^Stock et al. [92], ^i^Dunne et al. [93, 94], ^j^Romanou et al. [28], ^k^Lerner et al. [95], ^l^Totterdell [96], ^m^Yool et al. [97], ^p^Watanabe et al. [30], ^q^Hajima et al. [98], ^r^Ilyina et al. [51, 52], ^s^Paulsen et al. [99], ^t^Nakano et al. [100], ^u^Tjiputra et al. [101], ^v^Tjiputra et al. [102]. Bold text highlights when a model component has been completely updated

Tables 1, 2 and 3 map the key updates made between CMIP5 and CMIP6 (full details are available in Table S1). Table 1 suggests that most of the changes have tried to address at least one missing process of major importance for marine biogeochemistry, as highlighted in IPCC AR5 ([2], page 499), that is, representation of the lower trophic level including bacteria, organic matter cycling including sinking particles or variation in stoichiometric ratios.

**Table 2:**
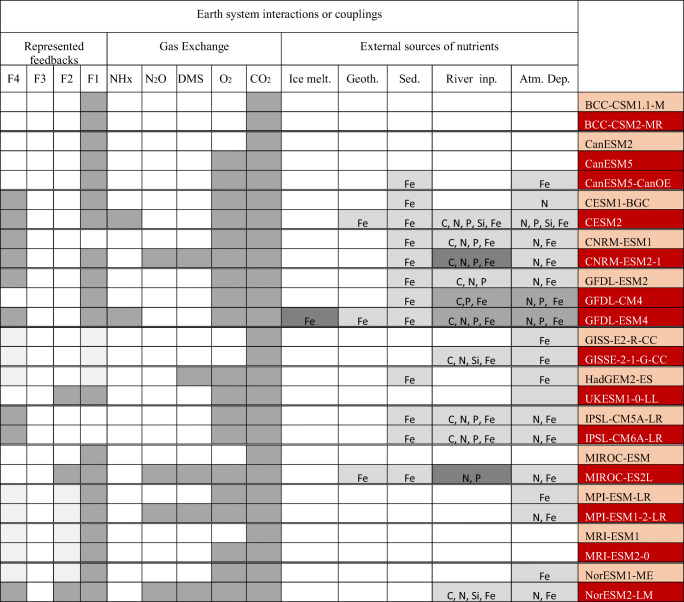
Overview of the ocean and marine biogeochemical components of Earth system models as used in CMIP5 and CMIP6. The names of the ESM are given in the first line of the table where the CMIP6 ESMs are given in red cells and the CMIP5 predecessors are given in pink cells. The Earth system interactions or couplings represented within the ESMs involving the marine biogeochemical models are described using three characteristics: the external inputs of nutrients or carbon-related fields conveyed by external boundary conditions are given with the chemical acronyms (C, P, N, Si, Fe), the representation of the gas exchange of greenhouse gases or reactive chemical species and the representation of Earth system feedbacks. In the last rows, ‘Fx’ indicates that the climate feedbacks or Earth system interactions ‘x’ as depicted in Fig. 1 is represented in a given Earth system model

**Table 3:**
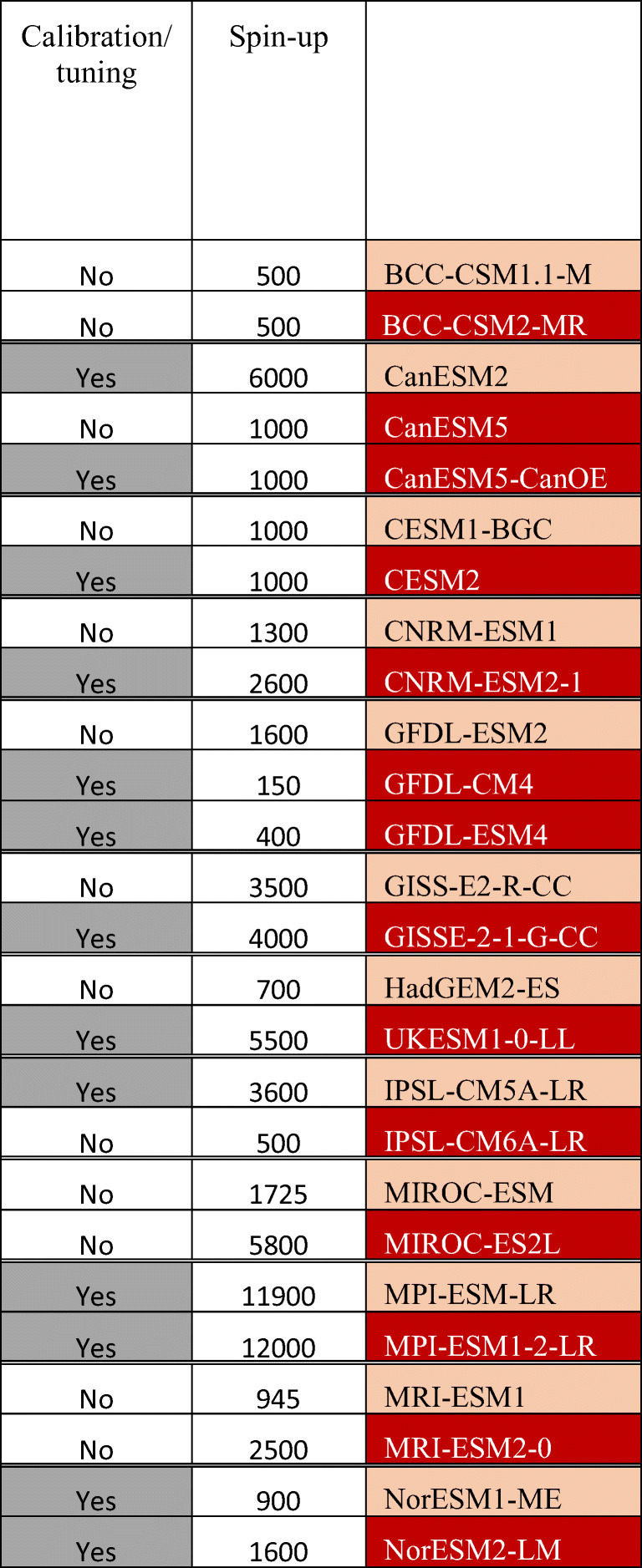
Overview of the ocean and marine biogeochemical components of Earth system models as used in CMIP5 and CMIP6. The names of the ESM are given in the first column of the table where the CMIP6 ESMs are given in red cells and the CMIP5 predecessors are given in pink cells. The modelling framework conducted by the various modelling groups for CMIP5 and CMIP6 is reviewed using two key characteristics: the duration of the spin-up simulation and the use of calibration/tuning procedure (further details about model calibration/tuning is given in Table S1)

Table 1 includes a brief overview of the key updates in ocean physics between CMIP5 and CMIP6 because marine biogeochemistry is prominently driven by ocean circulation (large-scale circulation and mesoscale eddies) and vertical mixing.

Table 1 tracks not only updates in the horizontal and vertical resolution of physical ocean models but also changes in related ocean physical parameterization. As suggested by Griffies et al. [103], an increase in horizontal or vertical resolution enables the representation of finer-scale ocean physical processes (e.g. mesoscale eddies) in relation with the activation of more realistic ocean physical parameterizations (such as vertical mixing, diurnal cycle or coupling with the atmosphere).

The first common difference between CMIP5 and CMIP6 ESMs comes from the ocean-sea ice components. Indeed, it is interesting to note that 8 ESM groups out of 12 use an upgraded version of the ocean models or employ a new ocean model (Table 1). These changes imply substantial updates or revisions in ocean physical parameterizations that may have an impact on large-scale circulation and vertical mixing.

In addition, another common difference between ocean models used in CMIP5 and CMIP6 is the grid resolution. It is interesting to note that all of the ocean models, with the exception of MPI-ESM1-2-LR, now resolve ocean dynamics at a minimum horizontal nominal resolution of 100 km. The highest horizontal nominal resolution in the available multi-model ensemble is 50 km (GFDL-ESM4). Despite this general increase in horizontal resolution, only GFDL-CM4 uses an eddy-permitting ocean model (~ 25 km). In addition, the current generation of ocean models also better represent vertical physical processes with a typically finer vertical resolution.

Another common difference between the two generations of models is the complexity of the marine ecosystem description and related parameterizations. Here, the complexity encompasses the diversity of model trophic web (i.e. the number of specific model phytoplankton and zooplankton types), the representation of bacteria, ecosystem functioning including macro- and micro-nutrient limitation (e.g. iron), and the variation in modelled stoichiometric ratios of carbon, nitrogen and other elements (e.g. photosynthetic pigment). Greater complexity does not necessarily imply a better representation of cycles and processes associated with each biogeochemical species, as it may introduce new degrees of freedom and/or non-linear (or at least not well controlled) interactions between parameterizations.

Table 1 shows that ocean biogeochemistry models span a wide range of complexity levels. The simplest models use ocean carbon cycle models based on the OCMIP protocol [20] that do not include marine biota or nutrients. Meanwhile, the most complex models include a broad trophic structure that groups marine organisms into plankton functional types based on their biogeochemical role, with mechanistic representations of nutrient limitation and variable stoichiometric ratios.

Table 1 also highlights noticeable changes in biogeochemical parameterizations between CMIP5 and CMIP6. They concern 10 biogeochemical models out of 12 reviewed in this study. These changes may be related to the change in model complexity or to a revised set of parameterizations (e.g. nitrogen fixation, remineralization, grazing, flux feeding; see Table S1).

We map updates and changes in ocean biogeochemical models along three major axes; axis 1. The trophic food web, the plankton internal physiology (e.g. variable stoichiometry, chlorophyll pigment) and nutrients cycling (iron cycle, nutrients cycles). This axis aims to track updates in biogeochemical dynamics and ecosystem functioning; axis 2. The external sources of nutrients; axis 3. The interactions of marine biogeochemistry with climate or ocean physics. The latter two axes track the level of integration of the marine biogeochemical model in the modelled Earth system.

It is important to stress that an increase or a decrease along one of those three axes does not necessarily imply an improvement in model performance or skill. In most cases, it reflects progress in process understanding (physical, biogeochemical or both), the inclusion of new Earth system interactions or the representation of climate feedbacks is required to investigate future scenarios.

Table 1 shows that the current generation of CMIP6 displays a greater diversity of marine biogeochemical models than CMIP5.

COBALTv2 (in GFDL-ESM 4), for instance, displays the highest trophic complexity level with 3 explicit phytoplankton classes, 1 implicit phytoplankton class, 3 explicit zooplankton classes and 1 explicit heterotrophic bacteria class; however, this model still employs a relatively simple parameterization of iron cycling. In comparison, PISCESv2-gas (in CNRM-ESM2-1) or PISCESv2 (in IPSL-CM6A-LR) includes 4 explicit plankton types (2 phytoplankton and 2 zooplankton), but two iron ligands and 5 iron forms [104]. MARBL-BEC (in CESM2) also includes an iron ligand and has opted for increasing ecosystem complexity by introducing variable C:P stoichiometry, based on PO_4_ concentrations [105], while maintaining 4 plankton types. It is interesting to note that, while limiting the number of nutrients, CanESM5-CanOE have evolved toward a more comprehensive treatment of marine biogeochemistry with 4 explicit plankton types and using variable stoichiometry [89]. In contrast with a general increase in complexity, NOAA-GFDL has started to use a reduced complexity marine biogeochemical model embedded in the high-resolution ocean model of GFDL-CM4. This approach implies a trade-off between computational costs and essential biogeochemical processes to represent the ocean carbon cycle as explained in Galbraith et al. [105]. Such diversity tends to mirror progress in the understanding of the impact of variable stoichiometric ratios on ecosystem dynamics and carbon assimilation by phytoplankton cells [106–110].

Table 1 shows that all CMIP6 models except GFDL-CM4 have evolved toward a more comprehensive treatment of elemental cycling including nitrogen, oxygen and iron cycling. This moderate increase in model complexity is supported by recent observations in phytoplankton functioning, nutrient limitation or plankton physiology [111–116] and the availability of a larger array of observational data (bio-ARGO and GEOTRACES) supporting the model evaluation and development (e.g. Tagliabue et al. [117]). On the other hand, this increase in complexity is also encouraged by the growing range of applications to which ESMs are being dedicated (e.g. marine resource applications as investigated in Lotze et al. [59] or Park et al. [64]).

Finally, Table 1 shows that all CMIP6 models have progressed toward a better representation of marine organic carbon cycling, sinking particles and marine sediments. In most cases, this component of marine biogeochemistry is parameterized using either a sediment box module or a meta-model based on downward fluxes of organic matter. Indeed, for several CMIP6 marine biogeochemical models, a more complex representation of sinking particles and organic matter pools (refractory classes or flux attenuation parameterization) replaces the generalized pools of organic matter used in the CMIP5 predecessors.

Table 1 also sheds light on noticeable changes in the representation of sediment interactions. Most of the reviewed CMIP6 ESMs now simulate this compartment with biogeochemical parameterization (e.g. balance, meta-model, sediment box) or with a comprehensive sediment module (12-layer sediments module).

Table 2 also shows that the representation of the external sources of nutrients (i.e. the third axis of our model complexity breakdown) has grown in complexity between CMIP5 and CMIP6. It mirrors a more comprehensive treatment of boundary conditions between ESM components (atmosphere, rivers, glaciers, etc.). Most of the current generation of ocean biogeochemical models now consider inputs of biogeochemical elements via atmospheric deposition or from rivers. The iron delivery from sediment mobilization, hydrothermal sources or ice melting is additionally considered by a small set of models. This reflects recent advances in understanding the global iron cycle [111–116]. In contrast, despite a better understanding of the role of submarine water discharge in ocean nutrient supply [118–121], this particular boundary condition is not considered in the current generation of ocean biogeochemical models.

Besides, it is interesting to note that a couple of CMIP6 ESMs now includes a more comprehensive treatment of interactions between the marine biogeochemistry and the other Earth system components. For instance, GFDL-ESM 4 simulates interactively most of the primary source of iron for marine biogeochemistry (atmospheric dust deposition, iceberg melting and river supply), enabling the representation of biogeochemical couplings observed in the real world (e.g. [122]).

Table 2 highlights that the current generation of ESMs displays a wider range of Earth system feedbacks or interactions. In our review, we have decomposed Earth system interactions involving marine biogeochemistry along two axes: (1) the air-sea exchange of greenhouse gases or reactive chemical compounds interacting with Earth’s radiative budget (and hence climate); (2) the represented Earth system interactions involving marine biogeochemistry (including the air-sea exchange of greenhouse gases or reactive chemical compounds and biophysical interactions); that is, what is really contributing to the Earth system model climate. This latter has been mapped into 4 feedbacks: climate-carbon cycle feedbacks (F1), biogenic aerosol-cloud feedbacks (F2), non-CO_2_ biogeochemical cycle feedbacks (F3) and phytoplankton-light feedbacks (F4).

The influence of ocean dimethylsulfide (DMS) emissions on cloud albedo is an example of the biogenic aerosol-cloud feedback (F2). DMS is a breakdown product of dimethylsulfoniopropionate (DMSP), a metabolite in many phytoplankton with a role as a cellular osmolyte/antioxidant [123, 124]. It is exchanged with the atmosphere and is involved in the formation of sulfur aerosols once it is oxidized there. As the other sulfate aerosols, DMS may be involved in the formation of cloud condensation nuclei (CCN). The potential importance of ocean DMS emissions for the climate system is still largely debated [125] because modern observations do not support its prominent role in the formation of CCN [126–128]. However, long-term measurement [129] and mesocosm experiments (e.g. [17]) suggest that global changes may impact the rate of ocean DMS emissions. Recent modelling studies argue for a potential role of ocean DMS in future climate change (e.g. [130, 131]). Ocean NH_x_ emissions are also involved in biogenic aerosol-cloud feedbacks (F2). Kirkby et al. [132] suggest that NH_x_ can also play an important role in the formation of secondary nitrate aerosols in the atmosphere. Similarly to DMS, these aerosols can serve as CCN and contribute to changes in cloud albedo. Non-CO_2_ biogeochemical cycle feedbacks (F3) involve ocean emissions of non-CO_2_ greenhouse gases (e.g. N_2_O or methane) or any chemical compounds contributing to the generation of greenhouses gases (e.g. methane, carbon monoxide). The phytoplankton-light feedbacks (F4) represent the suite of biophysical mechanisms that involve the influence of the marine biota on the upper ocean physics through the vertical redistribution of heat.

**Fig. 1:**
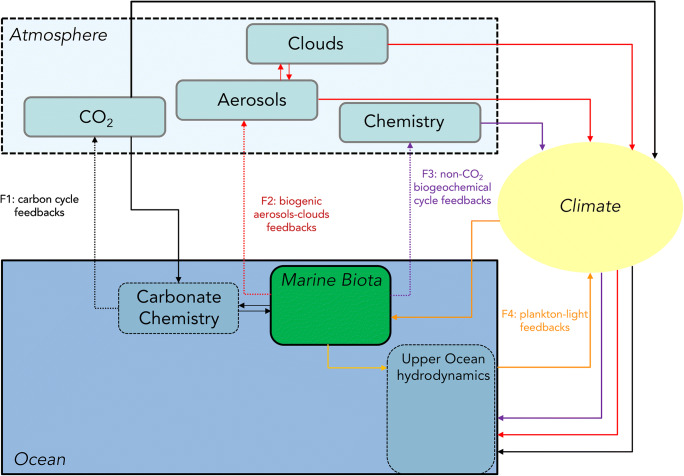
Schematic representation of Earth system interactions and feedbacks between the ocean biogeochemistry and climate. F1 represents the well-established climate-carbon cycle feedbacks; F2 and F3 sketch the dominant pathways for the biogenic aerosol-cloud feedbacks and the non-CO_2_ biogeochemical cycle feedbacks; F4 depicts the phytoplankton-light feedbacks (that is a biophysical interactions)

Table 2 confirms that all ocean biogeochemical models account for the climate-carbon cycle feedback since CMIP5 (Earth system feedback F1 in Fig. 1). In addition, Table 1 shows that the current generation of ocean biogeochemical models includes an air-sea gas exchange for a larger number of radiatively active biogeochemical compounds such as DMS, nitrous oxide (N_2_O) and ammonia (NH_x_). The inclusion of climate active gases or greenhouse gases other than CO_2_ in the current generation of ocean biogeochemical models is a result of the increased recognition of the importance of these compounds in Earth system interactions with aerosols, atmospheric chemistry and, potentially, with clouds.

In particular, the inclusion of ocean NH_x_ or N_2_O emissions in ocean biogeochemical models has been driven by a better understanding of the global nitrogen cycle and its role in climate change. In particular, the development of databases such as MEMENTO (https://memento.geomar.de/) has enabled better validation and calibration of N_2_O modules in global ocean biogeochemical models [133–138].

However, the inclusion of Earth system feedbacks as illustrated in Fig. 1 has not in all cases progressed between CMIP5 and CMIP6. For example, biophysical interactions with the ocean radiative transfer (F4 in Fig. 1) are overlooked by more than half of the marine biogeochemical models examined, although this feedback is well documented and relatively well understood [139, 140].

Our review of available ESMs suggests that the current generation of marine biogeochemical models has not much evolved toward comprehensive couplings between Earth system components and ocean biogeochemistry or toward improved treatment of biophysical and biogeochemical feedback with respect to their predecessors (F1 and F4 in Fig. 1). The full impact of ocean biogeochemistry on climate and its role in Earth system feedback remains far from being entirely represented in the current generation of Earth system models, as it involves different spatial and temporal scales that models are not currently able to reach and also processes still poorly understood.

Finally, our review suggests that the modelling approaches have evolved between CMIP5 and CMIP6. These latter have been monitored with two key indicators: (1) the length of the spin-up simulation and (2) the use of calibration/tuning for marine biogeochemical parameters. These two key indicators were discussed in published literature (e.g. Séférian et al. [76] or Hourdin et al. [141]), reflecting, in general, an improved knowledge in model characteristics (strength and deficiency).

Table 3 and Table S1 highlight that most of the modelling groups have expanded the duration of the spin-up for CMIP6. This represents an important effort of the scientific community to converge toward recommended standards (e.g. [142]). Only GFDL and IPSL have reduced the duration of their spin-up protocol for computing reasons: they manage to fulfil CMIP6 standard in a few hundreds of years. On the other hand, it is noticeable that several modelling groups have included a step of model calibration or tuning in CMIP6. Our review suggests that this step has been motivated by various reasons: bias reduction for key biogeochemical fields in CNRM, GFDL or NorESM or bias compensation to reduce the impact of known biases in simulated surface chlorophyll for ocean DMS and organic aerosols emissions in UKESM. There is no consensus between modelling groups on how model calibration or tuning takes place in the model preparation. Depending on modelling group, the calibration or tuning is either included in the model development or during the spin-up procedure (Table S1).

## Tracking Model Performance Across Two Generations of Models

**Fig. 2:**
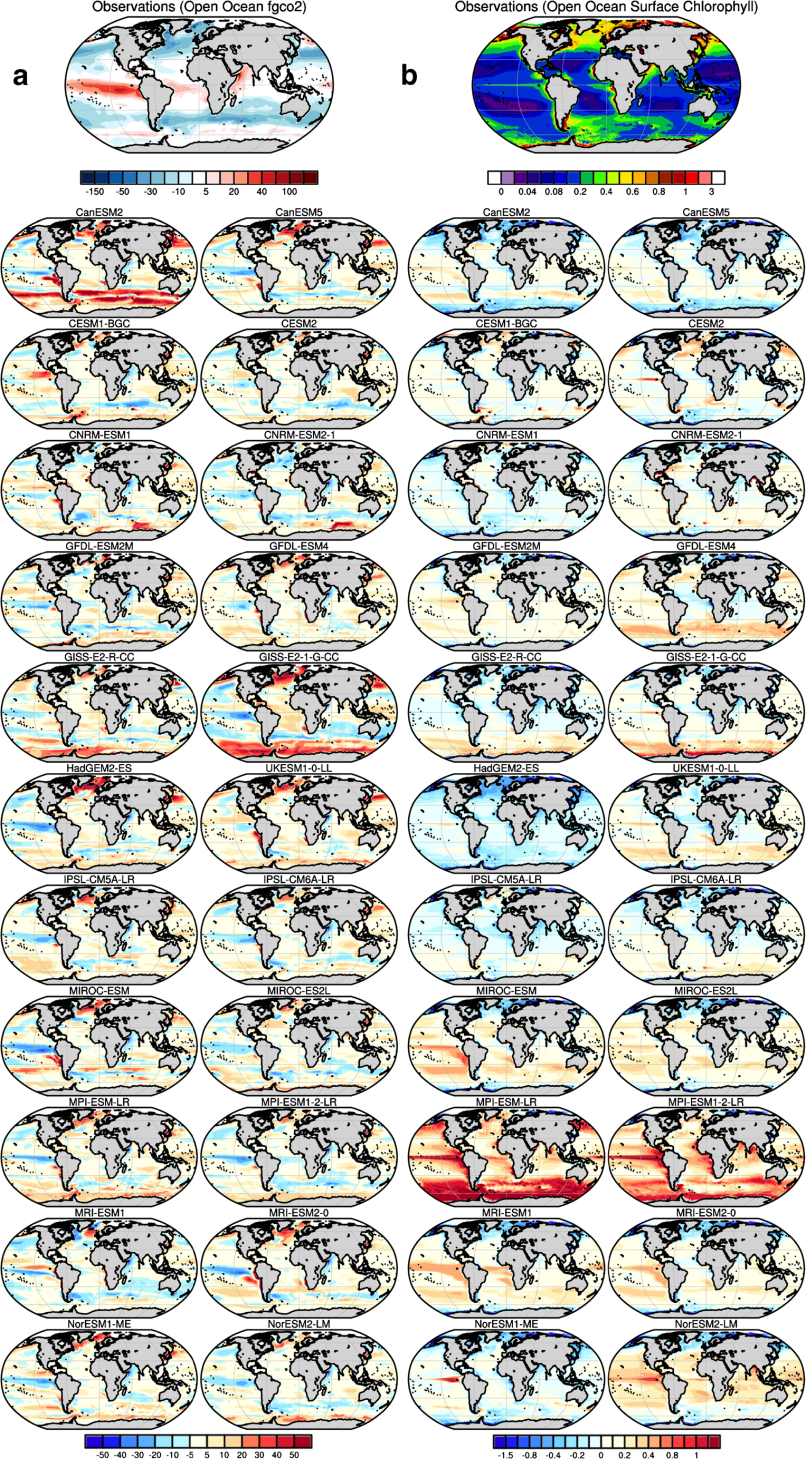
Model-data intercomparison of **a** open ocean-sea-air carbon fluxes (fgco2, g C m^−2^ year^−1^) and **b** open ocean surface chlorophyll (chl, mg Chl m^−3^) as simulated by ocean biogeochemical models embedded within CMIP6 Earth system models (the right column) and their former version as used for CMIP5 (the left column). **a** The first top panel shows observation-based estimates from Landschützer et al. [143] averaged for the period 1995–2014 (see Methods in Supplementary materials). The other panels show model-data biases averaged for the same period. Coloured areas are indicative of the model-data absolute difference in magnitude of sea-air fluxes. Red regions indicate areas in models where the magnitude of the sea-air flux is greater than that observed, whereas blue regions indicate the reverse. **b** The first top panel shows satellite-based ocean chlorophyll estimates from ESA-CCI-OC [144] averaged over 1998–2014. The other panels show model-data departure averaged over the period 1998–2014

**Fig. 3:**
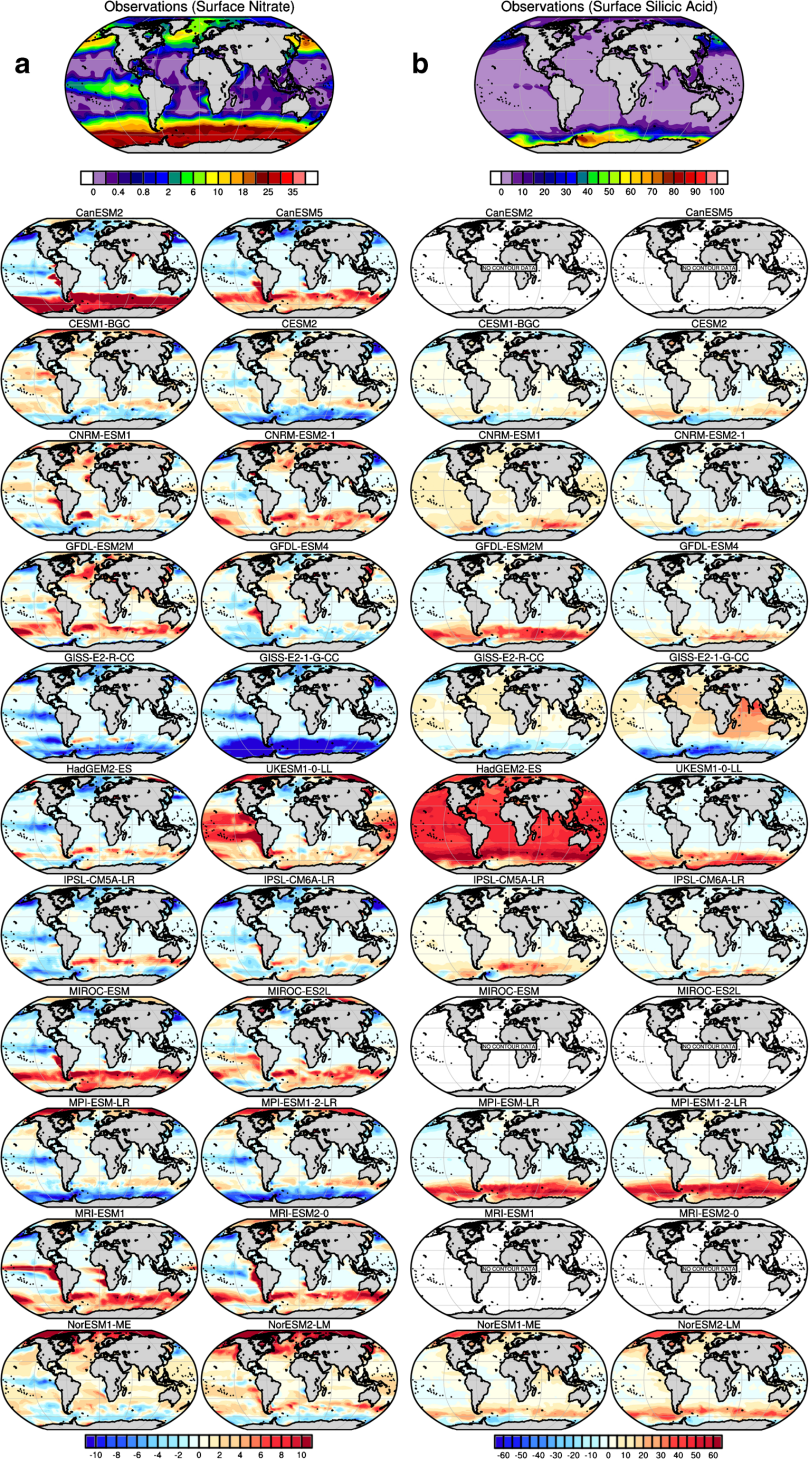
Model-data intercomparison of **a** surface nitrate concentrations (no3, μmol L^−1^) and **b** surface silicic acid concentrations (si, μmol L^−1^) as simulated by ocean biogeochemical models embedded within CMIP6 Earth system models (right columns) and their former version as used for CMIP5 (left columns). **a** and **b** The first top panel shows the optimal interpolation of nitrate (no3) and silicate (si) measurements as provided in the World Ocean Atlas Database 2013 (Garcia et al. [145]). The other panels show model-data departure averaged over the period 1995–2014 (see Methods in Supplementary materials)

**Fig. 4:**
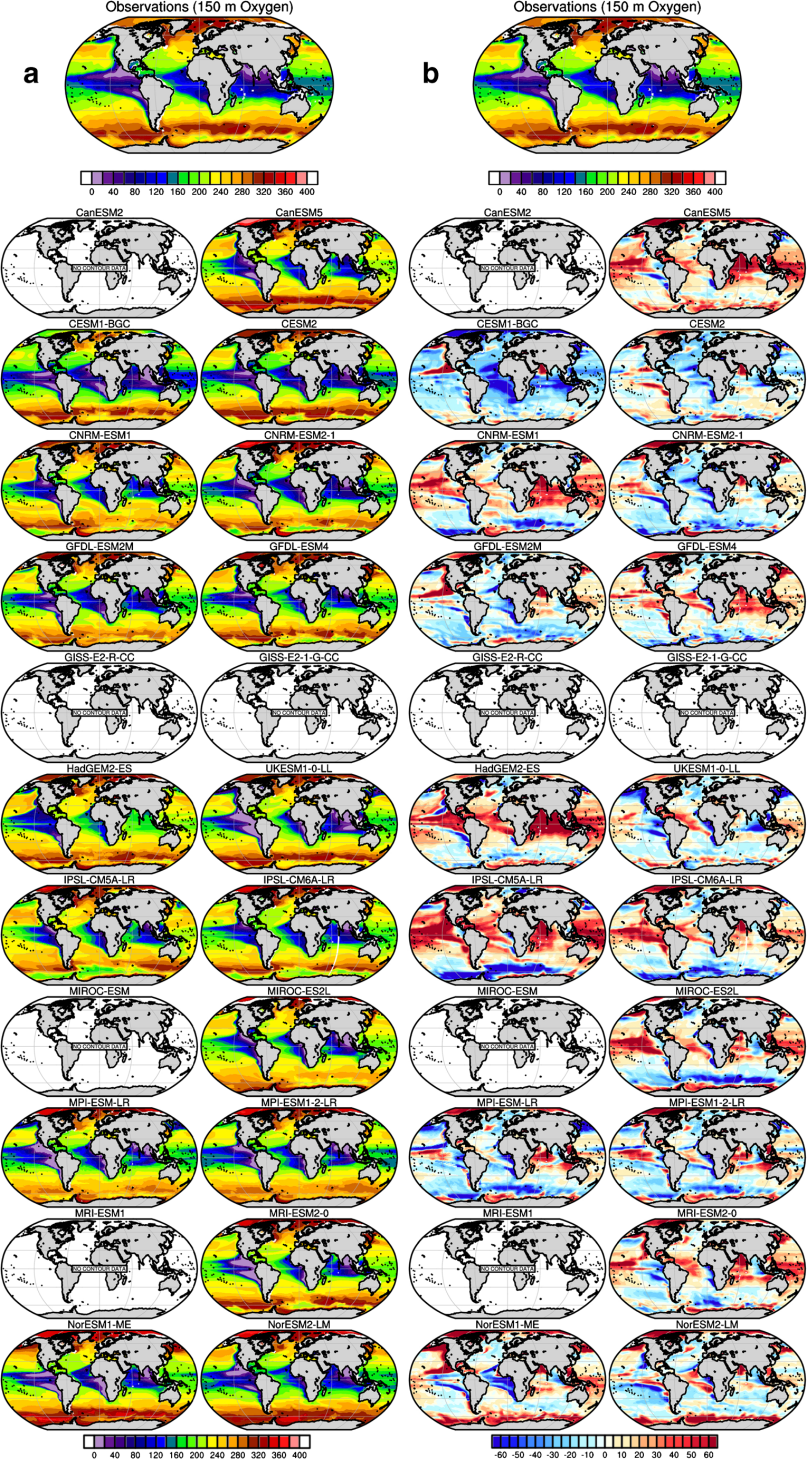
Model-data intercomparison of oxygen concentrations at 150 m (o2, μmol L^−1^) as a proxy for oxygen minimum zones (OMZs) and as simulated by ocean biogeochemical models embedded within CMIP6 Earth system models (on the right column) and within their former version used for CMIP5 (on the left column). The first top panels in **a** and **b** show the observed oxygen concentrations at 150 m from the World Ocean Atlas 2013 (Garcia et al. [145]). The other panels in **a** show oxygen concentrations at 150 m as simulated by CMIP5 and CMIP6 models averaged over the period 1995–2014, while panels in **b** show model-data departure averaged over the period 1995–2014 (see Methods in Supplementary materials)

**Fig. 5:**
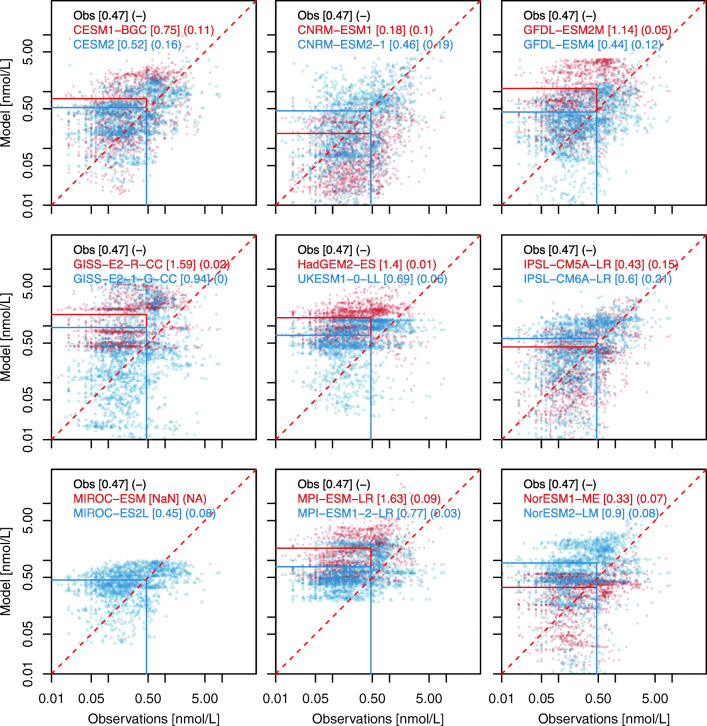
Model-data scatterplots for surface dissolved iron concentrations (log-log scale). Observational data are derived from the average of the 0–10 m of the measurement compilation used in Tagliabue et al. [117]. Model concentrations are taken from the first ocean layer. Red dots and blue triangles indicate CMIP6 and CMIP5 models respectively. The red dashed line shows the 1:1 line; the red and blue solid lines highlight the model-data mismatch in terms of global mean concentrations for CMIP5 and CMIP6 models (see Methods in Supplementary materials). The global mean for observations and models are given in brackets. Model-data fit (squared correlation, *R*^2^) is given in parenthesis with squared correlation coefficients for CMIP5 and CMIP6 models

Figures 2, 3, 4 and 5 illustrate the performance of the current generation of ESMs taking part in CMIP6, together with their predecessor CMIP5 models, for a range of climatological biogeochemical properties that are central to the carbon cycle and ecosystem applications: the sea-to-air flux of CO_2_, ocean chlorophyll, nitrate, silicate, oxygen and iron (see Methods in Supplementary materials). For Figs. 2, 3 and 4, observation-based estimates of each property are shown at the top of the figure, followed by the biases found across the current and last generation models. We note that, in several cases, observation-based estimates are derived from significant processing of sparse observations or from algorithms relating the quantity of interest to directly observed quantities (e.g. sea-to-air CO_2_ flux, satellite chlorophyll). As such, the observations themselves are also subject to uncertainty which will be discussed in the context of each comparison.

In Fig. 2a, the sea-to-air flux of the critical greenhouse gas, CO_2_, is shown, with a data product based on the mapping of observational pCO_2_ data drawn from the Landschützer et al. [143] product (1995–2014). The key geographical features of this are strong outgassing (i.e. a net sea-to-air flux) in upwelling regions, most clearly in the tropics and along the equatorial region of the Pacific Ocean, and ingassing (i.e. a net air-to-sea flux) at temperate and subpolar latitudes. These features reflect processes that are governed by temperature, patterns of deep water formation, surface biological production and the thermohaline circulation.

In general, both CMIP5 and CMIP6 generations of models show a mixture of positive and negative biases across the globe with disagreement in the sign of the carbon fluxes over some regions. Common patterns are slightly negative biases both in the equatorial Pacific (i.e. weak outgassing) and in the North Atlantic (i.e. excessive ingassing). Both generations of models show a mix of relatively small positive and negative biases, except for the CMIP5 CanESM2 which shows the largest model-data error across the model ensemble. However, the comparison with observations has been substantially improved in CanESM5. More generally, Fig. 2a highlights that the improvement in simulated sea-to-air carbon flux is clearer when looking at the direction of the carbon flux. This improvement seems to be linked to an improved representation of ocean vertical mixing (see skill scores of the ocean mixed-layer depth below). Indeed, all CMIP6 models exhibit smaller domains where the direction of the sea-to-air carbon flux disagrees with observations, except for MPI-ESM1-2-LR, which used the same ocean model and displays the same pattern of model-data disagreement for CMIP5 and CMIP6.

Figure 2 b shows surface chlorophyll, compared with satellite-based estimates derived from ESA-CCI-OC ocean colour data [144]. The key geographical features are relatively high concentrations in productive temperate, subpolar and upwelling regions, and extremely low concentrations in the unproductive subtropical gyres. The latter are dominated by perennially low-nutrient conditions, while the former experience frequent, or seasonal, introduction of nutrients by upwelling or deep mixing. While these general biome scale patterns are robust across satellite algorithms, we note that estimates diverge in the Southern Ocean [146], where global satellite-based chlorophyll algorithms have been found to significantly underestimate observations [147].

Several CMIP6 models compare more favourably with observations than their CMIP5 predecessors. All models displaying a pattern of generally negative bias in CMIP5 now exhibit large areas of both small positive and small negative biases. Models overestimating surface chlorophyll concentrations in CMIP5 now display reduced biases (< 0.4 mg Chl m^−3^). This improvement is small for MPI-ESM1-2-LR, which still overestimates surface concentrations of chlorophyll. Some CMIP6 models, such as CESM2, GISS-E2-1-G-CC and NorESM2-LM, display on the contrary larger model-data errors than their predecessors. Given the large diversity across the models, it is difficult to determine whether changes in physical ocean models or changes in ocean biogeochemical models are behind these changes.

However, it is interesting to note that three CMIP6 models (CNRM-ESM 2-1, IPSL-CM6A-LR and UKESM1-0-LL), which share a common ocean physics model, overlap in their patterns of positive and negative biases in spite of differences in marine biogeochemistry submodels (spatial correlation of model-data errors R^2^ = ~0.5).

It is notable that most of the models reviewed here overestimate surface chlorophyll estimates in the Southern Ocean. This bias, however, is likely due in part to the underestimation of Southern Ocean chlorophyll by the global satellite chlorophyll algorithms [147]. The substantial positive Southern Ocean bias in GFDL-ESM 4, for example, is significantly diminished when compared against Johnson’s Southern Ocean-specific satellite-based chlorophyll algorithms (e.g. [148]).

Figure 3 a and b show the distribution of surface nitrate (NO_3_) and silicic acid (H_4_SiO_4_), which are represented in both CMIP5 and CMIP6 models. Figure 3 a shows that only GFDL, IPSL and MIROC models have consistently improved their mean states between CMIP5 and CMIP6 for nitrate concentrations. In some cases, model generations show the same spatial patterns of biases, while others, most noticeably UKESM1-0-LL (where entirely new marine biogeochemistry has been incorporated), show a large overestimation of surface nitrate concentration over the tropics.

A comparison of simulated surface concentrations of silicic acid with modern observations shows that all models except GISS and CESM models have improved their representation of the surface distribution of silicic acid (Fig. 3b). The most striking improvement is seen between HadGEM2-ES and UKESM1-0-LL. Such an improvement is explained by the switch in the biogeochemical model component between CMIP5 and CMIP6, from Diat-HadOCC to MEDUSA-2.0 (see [96], for further details). Figure 3 b sheds light upon another systematic bias in the Southern Ocean where all the models display large model-data errors independent of their generation. It suggests that processes other than ocean resolution or the complexity of the marine biogeochemical model may be at the origin of this systematic model deficiency. The pattern of error differs among models. UKESM1-0-LL, MPI-ESM 1-2-LR and GISS-E2-1-G-CC display a uniform bias in simulated silicic acid concentrations, whereas all the other models show a mixture of positive and negative biases in simulated concentrations.

Figure 4 a presents the pattern of oxygen concentrations at a depth of 150 m where the signature of the oxygen minimum zone (OMZ) is expected to be visible. Note that 9 of 12 models simulated O_2_ in CMIP5, and one further model added O_2_ for CMIP6.

In general, CMIP6 models improve upon their CMIP5 predecessors in their representation of oxygen at 150 m (Fig. 4b). Model errors in the Southern Ocean have been reduced in CMIP6 with respect to CMIP5, highlighting a better representation of the deep ocean ventilation in the Southern Ocean or more accurate biogeochemical characteristics of outcropping water masses. Model-data errors have also been reduced in CMIP6 in large domains of the Indian Ocean where large OMZs occur although all models display a systematic overestimation of oxygen at 150 m in the Arabian Sea. The same feature is also observed in the tropical Pacific where a model-data error has been reduced in CMIP6 with respect to CMIP5. Contrasting with the other ocean domains, models’ performance has not improved in the Atlantic Ocean. For example, in the tropical Atlantic, some models have shifted in the sign of the model-data errors: from a negative bias in CMIP5 (stronger-than-observed OMZ) to a positive bias in CMIP6 (weaker-than-observed OMZ) or the opposite. In both cases, the absolute magnitude of the model-data errors in this region remains similar between model generations. This implies a systematic bias in ocean biogeochemical models which seems independent from ocean resolution or complexity of marine biogeochemistry models. Besides, our review of model performance highlights that open ocean hypoxia remains poorly represented in ocean biogeochemical models; the CMIP6 models still tend to overestimate this marine biogeochemical feature with respect to their CMIP5 predecessors. This is especially clear in the southern tropical Pacific, where all models except CESM2 and GFDL-ESM 4 overestimated the level of hypoxia of the OMZ (Fig. 4).

Improvement in GFDL-ESM 4 is explained by a suite of updates and changes in model physics (i.e. mixing and Southern Hemisphere climate) and biogeochemical parameterizations (i.e. the use of a revised remineralization scheme for organic matter depending on oxygen and temperature of Laufkötter et al. [148]). In addition, COBALTv2 has lower net primary productivity than TOPAZv2 which allows the high-nutrient low-chlorophyll region to spread further meridionally in the tropical Pacific and reduce the eastern equatorial nutrient trapping and associated oxygen decline.

The surface distribution of dissolved iron is also an important feature of marine biogeochemistry. Its availability controls marine biological production in several ocean regions [149]. As for oxygen, Table 1 highlights that marine iron cycling is not represented in all biogeochemical models. Nonetheless, this number has increased in CMIP6 (Table 1). It translates the current scientific consensus which recognizes the need to resolve the iron cycling in biogeochemical model in order to better simulate the marine biogeochemical dynamics, e.g. for glacial-interglacial climate change [150] or for variability and response to climate change [151].

Figure 5 illustrates, however, that the performance of the current generation of models with respect to iron does not improve much with respect to that of the previous generation. Indeed, the model-data fit estimated with squared correlation coefficients remains < 0.25. This fit has not progressed much from CMIP5 to CMIP6, except possibly for IPSL and CNRM models which both employed PISCESv1 [40, 41] for CMIP5 and PISCESv2 [91] for CMIP6. As highlighted in Aumont et al. [91], PISCESv2 includes a more detailed representation of the ocean iron cycle compared with PISCESv1.

The poor agreement between the observed and simulated distribution of dissolved iron relative to macronutrients (Fig. 3) partly reflects differences in the nature of the datasets. The relatively large number of nitrate measurements globally, for example, has allowed construction of robust climatological patterns [145] that model climatologies can be compared against. The relative paucity of dissolved iron measurements, in contrast, requires a comparison of modelled climatologies against patchy individual measurements. Despite this, Fig. 5 shows that some CMIP6 models better simulate the global average concentration of dissolved iron than their predecessors. This is particularly clear for UKESM1-0-LL, MPI-ESM 1-2-LR and GFDL-ESM 4. It is interesting to see the various modelling approaches for representing marine iron cycling. UKESM1-0-LL and MIROC-ES2L, for instance, use respectively Dutkiewicz et al. [152] and Moore and Braucher [153] parameterization for marine iron cycling that removes dissolved iron concentrations above an ad hoc threshold. Other ocean biogeochemical models use mechanistic iron cycling schemes that avoid the needs of ad hoc thresholds (e.g. PISCES-v2 and PISCES-v2-gas employs Völker and Tagliabue [154] formulation and TOPAZv2 applies an empirical relationship to dissolved organic carbon (DOC) to derive ligand concentrations).

Table 4 provides a large-scale picture of the model’s ability to simulate key downward biogeochemical fluxes involved in global carbon and nutrients cycling. Most of the CMIP6 marine biogeochemical models better simulate the magnitude of the surface and 100 m biogeochemical fluxes than their CMIP5 predecessors. Indeed, CESM2, CNRM-ESM2-1, GISS-E2-1-G-CC, IPSL-CM6A-LR, MPI-ESM 1-2-LR and NorESM2-LR have improved the representation of at least one biogeochemical fluxes with respect to their CMIP5 predecessors; BCC-CSM2-MR, CanESM5, GFDL-ESM 4 and MIROC-ES2L display comparable performance; only CanESM5-CanOE, MRI-ESM 2-0 and NorESM2-LM have respectively degraded the representation of either the vertically integrated net primary productivity or the carbon export at 100 m compared with their CMIP5 predecessors.

**Table 4:**
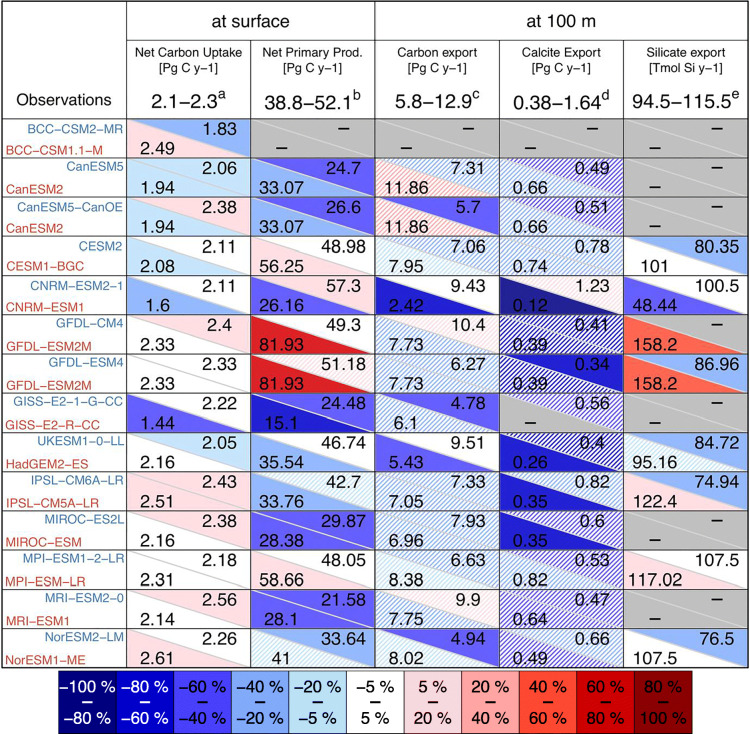
Comparison between observational and model estimates of biogeochemical fluxes over the modern period. For both CMIP5 and CMIP6 models, biogeochemical fluxes are calculated over the 1995–2014 period (see Methods in Supplementary materials)

Observational estimates are derived from the following database: ^a^Landschützer et al. [143] product average over 1995–2014 and adjusted for the pre-industrial ocean source of CO_2_ from river input to the ocean consistently with the methodology employed in [155] that used a river flux adjustment of 0.78 Pg C year^−1^ [156]; ^b^maximal range of remote-sensing estimates from Behrenfeld et al. [157] and Kulk et al. [158]; ^c^Dunne et al. [159] and ^d^Tréguer and De La Rocha [160]. When required, the modelled net ocean carbon uptake is corrected with the net riverine-induced outgassing diagnosed from the piControl simulation. Coloured cells indicate the relative deviation in model global estimates with respect to the observation median best estimates; hatched coloured cells indicate where model global estimates fall within the observational uncertainty range. Grey cells indicate missing or unrepresented biogeochemical fluxes

Despite the general improvement, Table 4 highlights that several CMIP6 models fall outside the range of remote-sensing estimates of primary production ([157, 158, 161]). It suggests that the current generation of marine biogeochemical models still has difficulties to model underlying processes involved in the carbon fixation by phytoplankton (such as nutrient colimitation, nitrogen fixation, remineralization), required to accurately simulate the magnitude of the vertically integrated net primary productivity. At the same time, it is also important to acknowledge that there are still large uncertainties in remote-sensing-based estimates of primary production, e.g. 38.8–42.1 Pg C year^−1^ in the most recent estimates of Kulk et al. [158] and 47.5–52.1 Pg C year^−1^ according to Behrenfeld et al. [157].

**Fig. 6:**
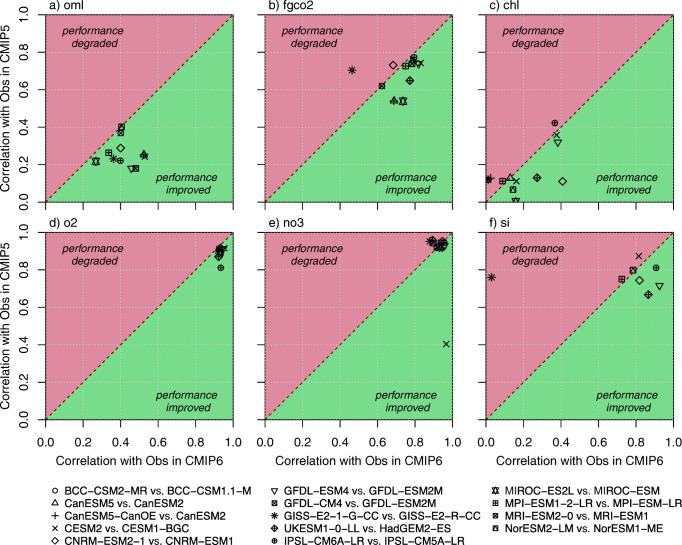
Scatter plot confronting the performance of CMIP6 models to replicate the geographical structure of observed fields with respect to that of their CMIP5 predecessors. The performance metrics are the model-data spatial correlation computed from yearly averaged data and model outputs. The variables of interest are mixed-layer depth (oml), air-sea CO_2_ flux (fgco2), surface chlorophyll (chl), oxygen concentration at 150 m (o2) and surface concentrations of nitrate (no3) and silicic acid (si). The green (red) shading flags an improvement (degradation) of the model performance to replicate the observed geographical structure for a given field. The ocean mixed-layer depth is computed similarly in all models; it is based on a density criterion of 0.03 kg m^−3^. The ocean mixed-layer depth simulated by the various Earth system models is evaluated against the observational dataset of de Boyer Montégut et al. [162]

**Fig. 7:**
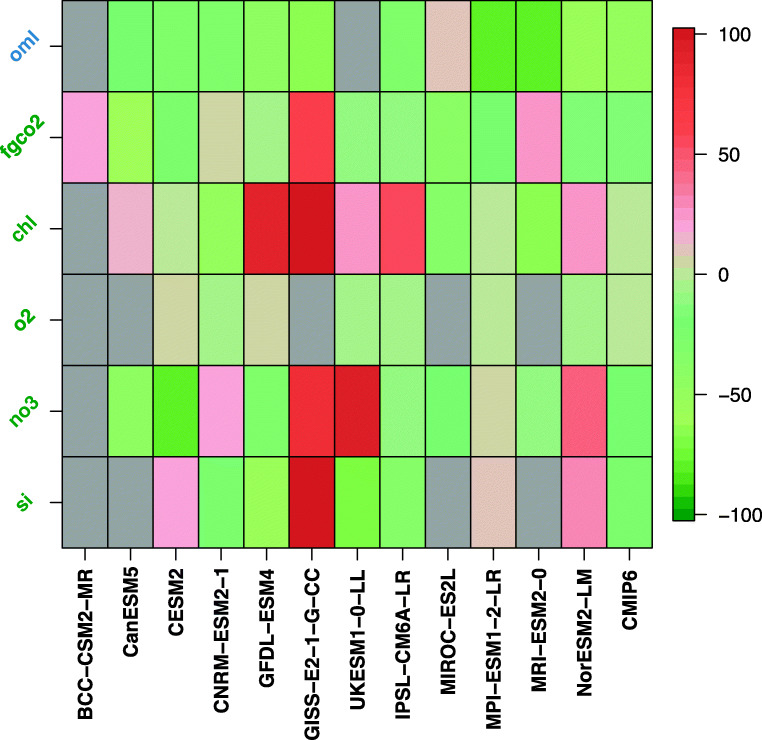
Portrait diagram highlighting the performance of CMIP6 models (one representative per modelling groups) with respect to their CMIP5 predecessors. The variables of interest are mixed-layer depth (oml), air-sea CO_2_ flux (fgco2), surface chlorophyll (chl), oxygen concentration at 150 m (o2) and surface concentrations of nitrate (no3) and silicic acid (si). The skill score metric, Z-score, is computed for a given model and for a given field as follows: Z-score$=\frac{{\mathrm{RMSE}}_{\mathrm{CMIP}6}(M)-{\mathrm{RMSE}}_{\mathrm{CMIP}5}(P)}{{\mathrm{RMSE}}_{\mathrm{CMIP}5}(P)}\times 100$, where RMSE_CMIP6_(*M*) is the global area-weighted average model-data root-mean-squared error (RMSE) of the model of the current generation contributing to CMIP6 and RMSE_CMIP5_(*P*) is the RMSE of its predecessor that has contributed to CMIP5. Greenish (reddish) colours and negative (positive) Z-scores indicate improved (degraded) field representations in CMIP6 model versions; darker colours indicate a greater change from CMIP5 to CMIP6. Grey indicates missing data for one or both generations of models. Air-sea CO_2_ flux (fgco2) was adjusted for riverine-induced outgassing as in Table 4. The ocean mixed-layer depth is computed similarly in all models; it is based on a density criterion of 0.03 kg m^−3^. The ocean mixed-layer depth simulated by the various Earth system models is evaluated against the observational dataset of de Boyer Montégut et al. [162]

Figures 6 and 7 track changes in performance between CMIP5 and CMIP6 marine biogeochemical models. Figure 6 highlights how far the CMIP6 models have improved their capability to simulate observed spatial patterns with respect to their CMIP5 predecessors; Fig. 7 summarizes the overall model performance including information on model performance to reproduce observed distribution (pattern and magnitude).

Both Figs. 6 and 7 show that CMIP6 models have improved the representation of the ocean physics (here the ocean mixed-layer depth). The cross-generation picture of the model performance for marine biogeochemistry is more contrasted. Globally, Figs. 6 and 7 show that most of the CMIP6 models outcompete their CMIP5 predecessors. However, this improvement remains modest. Except for some models displaying a noticeable improvement for one or two biogeochemical fields (surface nitrate for CESM2, surface chlorophyll for CNRM-ESM2-1, surface silicic acid for GFDL-ESM 4), most of the CMIP6 model display a slight increase in model-data spatial correlation (up to + 0.2, Fig. 6) or an overall reduction in model-data RMSE of about 20% (Fig. 7). Besides, this improvement does not concern all models. For instance, GISS-E2-1-G-CC shows a noticeable degradation in performance for all of the biogeochemical fields analyzed here.

## Conclusions

### Summary of 5 Years of Ocean Biogeochemical Model Development

Our review of available Earth system models highlights that the current generation of marine biogeochemical models used for CMIP6 displays a greater diversity than the previous one used for CMIP5. Several marine biogeochemical models have evolved toward a more comprehensive representation of marine biogeochemistry (i.e. CESM, CNRM, GFDL, IPSL, MIROC, UKESM), typically including an expanded array of biological taxa (e.g. diazotrophs) or elemental cycling (e.g. oxygen and iron cycles), variable stoichiometry, sediments (e.g. sediment box module) and the representation of (non-CO_2_) trace gases relevant to atmospheric chemistry. On the opposite, some groups have limited the increase in model complexity between CMIP5 and CMIP6 (i.e. BCC, GISS, MPI, MRI, NorESM). Finally, it is interesting to note that some groups have started to investigate the use of reduced complexity marine biogeochemical model (i.e. GFDL) or to intercompare in a traceable framework the impact of rising complexity on the simulated marine biogeochemistry (CanESM).

When assessed against observations, most of the CMIP6 models generally outperform their CMIP5 predecessors in many regions and for most of the marine biogeochemical fields reviewed here (Figs. 6 and 7 and Table 4). However, this model review has also highlighted several systematic model-data errors that are persistent even in CMIP6 models (e.g. oxygen concentrations at 150 m in tropical Atlantic, nutrient trapping in the Southern Ocean).

Our review also shows that the modelling approaches have evolved between CMIP5 and CMIP6. Indeed, most modelling groups have spun-up their model over a longer period for CMIP6 with respect to CMIP5 in order to fulfil the drift criterion as proposed by Jones et al. [142]. In contrast, the use of tuning and calibration for marine biogeochemical models for CMIP remains a less common feature at the time of CMIP6.

Finally, our review of model mean state performance against their model properties (resolution, complexity) suggests that neither increasing resolution nor increasing complexity leads automatically to model improvement. Instead, improvement is a mixture of improved ocean physical processes and better representation of biogeochemical processes.

In the context of improving confidence in future climate projections, it is important to stress that the model mean state performance is not the only mean to understand multi-model uncertainty, comparisons against seasonal to multi-annual variations in observed quantities may ultimately prove most critical to building confidence in future climate projections (e.g. [13, 163]).

### What’s Next?

In this final section, we identify some directions where marine biogeochemical models could continue to improve or to progress.

The first step change to expect in the next generation of models is the emergence of high-resolution ocean biogeochemical models fit to investigate centennial-scale simulation. This step change may be supported in a number of ways: (1) the availability of greater computational resources; (2) the use of hybrid-resolution numerical schemes to decrease the cost of biogeochemical models (e.g. [164]); (3) actually reduced complexity of marine biogeochemical models (e.g. such as miniBLING; [105]); (4) the use of machine learning to either accelerate marine biogeochemical models or to reduce the numerical cost necessary to improve their performance (i.e. via tuning). These (and potentially other) step changes will help to understand the extent to which mesoscale or sub-mesoscale ocean physics might change the response of marine biogeochemistry to rising CO_2_ and climate change—a missing factor in such models already highlighted from CMIP5 and IPCC AR5 [2].

A second important step change is related to the phytoplankton physiology and evolution. This change may have two benefits. First, several recent studies show that the inclusion of a more comprehensive treatment of plankton physiology may improve model performance, in particular some systematic biases in the Southern Ocean (e.g. [108, 165]). Then, this improvement is arguably a first step toward the representation of adaptation and fitness in ocean biogeochemical models [166, 167]. This omission remains an important caveat for multi-stressors studies (e.g. [9]) or time-of-emergence studies [168] as current models effectively assume no change in the underlying properties of modelled plankton.

Future developments should be pursued in the context of the internal cycling of micronutrients involved in phytoplankton physiology and metabolism such as iron, zinc or copper. Our review confirms that the current generation of marine biogeochemical models are still struggling to reproduce the major features of the oceanic iron distribution although the observations of dissolved iron in the ocean are growing rapidly [149] and are made widely available by GEOTRACES [169]. A key challenge for iron is that the dissolved iron commonly measured only appears to represent a trace residual of the underlying fluxes [170], pointing to the need for more process studies and observations of fluxes. It is possible that iron isotopes may yield further insight into the role of external inputs and internal cycling in shaping iron distributions in the observations and models. Finally, the development of additional model components dealing with other trace metals, such as cobalt [171], zinc [172], manganese [173] and copper [174], may also prove beneficial in constraining the magnitude and dynamics of external inputs in particular.

An expanded array of biological taxa may also be expected in the next generation of ocean biogeochemical models. A potentially important change in the ocean ecosystem modelling paradigm is the inclusion and integration of mixotrophs which are an important grazer of bacterioplankton, and which also feed on phytoplankton, microzooplankton and (sometimes) mesozooplankton. Mixotrophic bacterivory among the phytoplankton may be important for alleviating nutrient stress and may increase primary production in oligotrophic waters. Some modelling studies indicate that mixotrophy has a profound impact on marine planktonic ecosystems and may enhance primary production, biomass transfer to higher trophic levels and the functioning of the biological carbon pump [175].

This expanded array of biological taxa may take the concept of the marine biogeochemical model up to the marine ecosystem model, which will enable the representation of feedbacks of the marine trophic food web on marine biogeochemical cycles. The work of Lefort et al. [57] provides an example of this type of marine ecosystem model realizing a comprehensive coupling between a marine biogeochemical model (PISCES) with a marine trophic food web model (APECOSM).

A third important step change is related to the couplings between Earth system components and ocean biogeochemistry. Our review highlights that models have evolved toward a more comprehensive treatment of biological boundary conditions (e.g. atmospheric deposition, riverine inputs, sediments, ice sheets, geothermal sources) but that these latter are currently largely represented using climatological data rather than dynamic connections. Progress toward more complete couplings between Earth system components such as rivers, ice sheet/iceberg calving and ice shelves or atmospheric aerosols can help to better simulate interactions between marine biogeochemistry, biogeochemical cycles and climate.

In the same manner, a more comprehensive treatment of biophysical and biogeochemical feedback could be realized in the next generation of marine biogeochemical models. The latter involves, for instance, ocean emissions of greenhouse gases or biogenic volatile organic compounds (BVOCs) that are already simulated by a small number of models (see Table 5). However, our understanding of the global cycles of DMS, N_2_O and CH_4_ (including, specifically, the processes that produce them) is much less developed compared with CO_2_. Therefore, better treatment of biophysical and biogeochemical feedback requires a larger array of observational data sets in order to improve our understanding of the processes underlying these ocean emissions.

**Table 5:** Ocean natural emissions of non-CO_2_ trace gases simulated by CMIP6 models

	DMS (Tg S year^−1^)	N_2_O (Tg N year^−1^)	NHx [Tg N year^−1^)
Observational estimates	17.6–34.4^a^	1.9–9.4^b^	2–5^c^
CNRM-ESM 2-1	24.38	3.97	-
GFDL-ESM 4	-	-	3.10
UKESM1-0-LL	16.19	-	-
MIROC-ES2L	18.46	4.31	-
MPI-ESM 1-2-LR	-	8.89	-
NorESM2-LM	20.0	-	-

^a^Lana et al. [176]; ^b^Buitenhuis et al. [177]; ^c^Paulot et al. [178]

From the perspective of tracking future model improvement, it is important to stress that our capacity to assess model performance resulting from any of the potential advances discussed above is contingent upon continued improvement in observational constraints. Existing constraints were adequate for detecting large skill differences between CMIP5 and CMIP6 models, but the overall improvement in models necessitates more precise comparisons to detect skill differences. Such comparisons are challenged by data sparsity and uncertainties in algorithms designed to derive global fields from sparse data or infer properties of interest from remotely sensed variables. Continued improvement in the quality and quantity of data-based constraints is critical.

That being said, our review of the available pairs of CMIP5-CMIP6 marine biogeochemical models strongly suggests that careful consideration is needed when selecting model complexity with regard to the fitness-for-purpose of models (i.e. carbon cycle feedbacks, multiple Earth system feedbacks, multi-stressors, adaptation and biodiversity). Indeed, when confronting model complexity against model mean state performance, our work suggests that complex models do not necessarily outperform simple models. This is consistent with the earlier study of Kwiatkowski et al. [179], which directly led to the choice of marine biogeochemistry model in UKESM1-0-LL, where across many Earth system relevant metrics, the simplest model performed best. In this sense, our review shows that simple models (e.g. OCMIP nutrient restoring or NPZD type) remain viable when investigating carbon cycle feedbacks, although more complex models do still permit a better linkage with the marine biodiversity or a broader array of feedbacks and potentially more realistic Earth system behaviour.

## Electronic supplementary material

ESM 1 (XLSX 11927 kb)
